# Can serum isotope levels accurately measure intestinal calcium absorption compared to gold-standard methods?

**DOI:** 10.1186/s12937-015-0065-5

**Published:** 2015-07-31

**Authors:** Andrew P. Vreede, Andrea N. Jones, Karen E. Hansen

**Affiliations:** 1 Department of Medicine, University of Wisconsin School of Medicine & Public Health, Suite 4124 MFCB, 1685 Highland Avenue, Madison, WI 53705-2281 USA; 2 Department of Pediatrics, University of Wisconsin School of Medicine & Public Health, Madison, USA

**Keywords:** Accuracy, Methods, Calcium absorption, Postmenopausal women, Stable calcium isotopes

## Abstract

**Background:**

Low fractional calcium absorption (FCA) contributes to osteoporosis but is not measured clinically, as the gold-standard method requires administration of two calcium tracers and a subsequent 24-h urine collection. We evaluated alternate methods to measure FCA, compared to the gold standard method.

**Methods:**

We administered two stable calcium isotope tracers (~8 mg oral ^44^Ca and ~3 mg intravenous ^42^Ca) with breakfast to 20 fasting post-menopausal women (Cohort 1) 59 ± 7 years old with vitamin D insufficiency. We measured subsequent calcium isotope concentrations in 24-h urine samples and serum collected 1, 3 and 5 h post tracer administration during an inpatient research stay. We assessed the candidate serum estimates in a second cohort of 9 women with similar characteristics. Methods of measuring FCA were compared using correlation coefficients and Bland-Altman tests.

**Results:**

FCA estimated from a 3-h serum sample correlated highest with the levels from the 24-h urine collection (ρ 0.78, *p* < 0.001), but explained only 58 % of the variance in FCA. The total variance explained by 3-h estimates improved to 61 % with incorporation of glomerular filtration rate (GFR). FCA estimates from the 3-h serum measurement were assessed in a second group of nine women (Cohort 2) 60 ± 7 years old. In this cohort, however, FCA estimated by 3-h serum isotope levels did not correlate with gold-standard FCA measurements, whether determined with (ρ 0.02, *p* = 0.97) or without GFR values (ρ 0.03, *p* = 0.93). By contrast, FCA in Cohort 2 correlated best with 5-h serum isotope levels (ρ 0.75, *p* = 0.02).

**Conclusions:**

We conclude that serum isotope levels correlate with true fractional calcium absorption, but do not reliably estimate FCA when analyzed using Bland-Altman tests, compared to gold-standard methods.

**Trial registration:**

ClinicalTrials.gov.Identifier: NCT00933244

## Background

Nearly half of postmenopausal women sustain an osteoporotic fracture [1] and low calcium absorption is a risk factor for hip fracture [2]. Postmenopausal women have surprisingly high variability in calcium absorption efficiency, ranging from <10 to >50 % [3, 4]. Calcium absorption is influenced by numerous factors including age, calcium intake, estrogen status, vitamin D stores, gastrointestinal disorders and genetic factors [5, 6]. Measurement of calcium absorption could be performed, to recommend interventions to increase absorption and reduce fracture risk. Unfortunately, clinicians do not routinely measure calcium absorption in patients with osteoporosis due to lack of an accurate, inexpensive and time-efficient method.

Administration of an oral and an intravenous calcium tracer, followed by analysis of the dose-corrected ratio of the two tracers in a subsequent 24-h urine collection, is the gold-standard method to measure true fractional calcium absorption (FCA) [7]. The intravenous tracer allows a more precise measurement of FCA that accounts for renal calcium recycling and intestinal calcium secretion. Although validated [7], the method is time consuming and costly. Therefore, many researchers administer a single oral calcium tracer and measure its serum level 1-6 h later, to estimate FCA [3, 8–11]. While more efficient, the single isotope method might be less accurate than the 24-h urine method. Peak plasma tracer levels can be influenced by intestinal calcium excretion and transit time, renal calcium recycling and volume of calcium distribution. In 1994, Yergey et al. [12] assessed spot serum isotope levels at multiple time points within 24 h of tracer administration and concluded, by Bland-Altman analysis, that spot serum levels were inaccurate and introduced up to 69 % error in estimates of FCA.

Nonetheless, several researchers reported that single isotope methods correlate well with FCA. However, most studies did not use the 24-h urine method as the gold-standard method by which to assess serum estimates of FCA. Additionally, correlation coefficients were used to analyze data, rather than the Bland-Altman test [13], which more rigorously assesses agreement between two methods of measurement. Moreover, only one study [8] employed two cohorts, one to test candidate methods and a second to validate the best candidate method. Finally, few studies evaluated serum methods in postmenopausal women, a population at greatest risk for osteoporosis and in whom measurement would be most relevant clinically.

We evaluated whether serum isotope levels collected 1, 3 and 5 h post-tracer administration could accurately measure FCA in the first 29 postmenopausal women randomized into an ongoing clinical trial. We also examined whether incorporation of other factors, such as demographic, physical, dietary or laboratory attributes, could improve the accuracy of serum tracer estimates of FCA. We evaluated methods in an initial cohort, and then assessed predictor equations in a second cohort of subjects.

## Methods

### Subjects

We recruited participants for the study, “Treatment of Vitamin D Insufficiency,” (clinicaltrials.gov NCT00933244) through newspaper advertisements and letters of invitation to University of Wisconsin (UW) employees and participants of a research registry. Eligible subjects had serum 25(OH)D levels between 35 and 67 nmol/L (14 and 27 ng/mL) and were ≥5 years post-menopausal, or ≥60 years old if they reported hysterectomy without bilateral oophorectomy. Women were excluded if ≥75 years old or if they reported hypercalcemia, nephrolithiasis, inflammatory bowel disease, malabsorption, chronic diarrhea, diabetes, osteoporosis or had a GFR <45 mL/min based on the MDRD equation [14]. Subjects were also excluded if they had used bisphosphonates, estrogen compounds, calcitonin, teriparatide, oral corticosteroids or anticonvulsants within the prior six months.

### Procedures

Potentially eligible subjects, based on phone interviews, attended a screening visit at the UW Clinical Research Unit (CRU). Subjects completed a food frequency questionnaire [15] to estimate total (dietary and supplemental) calcium intake; subjects who agreed to consume 600-1400 mg of calcium/day were eligible for the study. Phlebotomy was performed to measure subjects’ serum 25(OH)D concentration via HPLC [16], serum calcium using cresolpthalein, albumin using bromocresol, creatinine using an IDMS-traceable methods and PTH using a chemilluminescent assay. Subjects who were eligible after the first visit underwent measurement of spine, hip and total body bone mineral density (GE Healthcare, Madison, WI); those with osteoporosis were excluded. The consent process included two verbal descriptions of the study (during the phone screen and first screening visit) followed by a written consent form which subjects were required to read and sign, prior to any study procedures. The study was approved by the UW Human Subjects Committee.

Eligible subjects completed a consecutive four-day food diary encompassing one weekend. The study nutritionist analyzed diet diaries using Food Processor software (ESHA Research, Salem OR, USA) to determine typical daily intake of energy, macronutrients, fiber, calcium, iron, magnesium, sodium, vitamin D, oxalate, caffeine and alcohol. The nutritionist designed each subject’s 24-h inpatient diet to replicate her typical outpatient consumption of nutrients.

To measure FCA using the gold-standard approach [7], subjects were admitted to the CRU at 0700 after fasting since midnight. Upon arrival, the subjects consumed breakfast containing a 300 mg calcium load, simultaneously drinking ≤50 mL of calcium-fortified orange juice containing ~8 mg of ^44^Ca and receiving ~3 mg of ^42^Ca intravenously over 5 min. The calcium isotope syringes were weighed before and after use to record the administered doses of ^42^Ca and ^44^Ca. Research nurses collected subjects’ urine for 24 h and blood samples 1, 3 and 5 h after isotope dosing. We chose to test these times because other researchers [2, 3, 11, 17] frequently estimated FCA using serum isotope levels at these time points. We calculated 24-h FCA using the Eastell formula [7]:

$$ FCA=\frac{\varDelta \%\ \mathrm{excess}\ {}^{44}\mathrm{C}\mathrm{a}\ \left(\mathrm{oral}\right)\ }{\varDelta \%\ \mathrm{excess}\ {}^{42}\mathrm{C}\mathrm{a}\ \left(\mathrm{intravenous}\right)}\times \frac{\mathrm{natural}\ \mathrm{a}\mathrm{bundance}\ {}^{44}\mathrm{C}\mathrm{a}}{\mathrm{natural}\ \mathrm{a}\mathrm{bundance}\ {}^{42}\mathrm{C}\mathrm{a}}\times \frac{\mathrm{dose}\ {}^{42}\mathrm{C}\mathrm{a}}{\mathrm{dose}\ {}^{44}\mathrm{C}\mathrm{a}} $$

Stable calcium isotopes (^44^Ca and ^42^Ca) were purchased from Trace Sciences (Wilmington, Deleware) as calcium carbonate powder; purity and enrichment were confirmed by high-resolution inductively coupled plasma mass spectrometry (HR-ICP-MS, ThermoFinnigan Element 2). The Waisman Clinical Biomanufacturing Facility reconstituted the calcium powders as previously described [18] and tested solutions for sterility and pyrogenicity. Personnel at the Wisconsin State Lab of Hygiene measured calcium isotope ratios using ^43^Ca as the internal standard (^42^Ca/^43^Ca and ^44^Ca/^43^Ca) in subjects’ serum and 24-h urine specimens by HR-ICP-MS, as previously described [18]. Briefly, each measured isotope ratio represented the average of five separate runs, each run consisting of 900 scans of the mass range of index. Precision of measurements were excellent, with an intra-assay and inter-assay coefficient of variation of 0.4 and 0.7 %, respectively.

### Statistical analysis

Data exhibited a normal distribution by normal probability plot and were summarized using the mean and standard deviation (SD). We used Spearman correlation coefficients (ρ), Bland-Altman tests and root mean square prediction errors to compare FCA determined from individual serum measurements to gold-standard measurements. For Bland-Altman analysis [13], a p-value >0.05 indicated no significant difference between paired values obtained using gold-standard and serum methods to measure FCA. Thus, a Bland-Altman p-value >0.05 would indicate that the two methods of FCA measurement are in agreement. We employed linear regression models to predict FCA based on subjects’ serum isotope levels, demographic, dietary and laboratory variables. Linear regression models were subsequently validated in Cohort 2. Statistical analyses were performed using R software (Version 3.0.1, The R Project for Statistical Computing, http://www.r-project.org) and Analyze-It (Version 3.15, Leeds UK). In all analyses, a p-value <0.05 was considered significant.

## Results

**Fig. 1:**
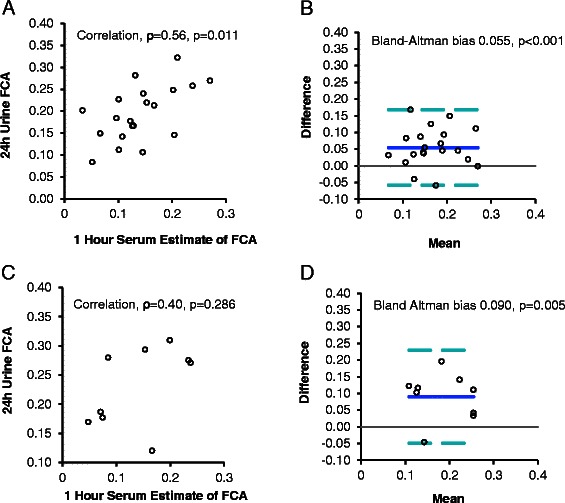
One Hour Serum Isotope Estimates Compared to 24-h Urine Fractional Calcium Absorption (FCA) Values. One-hour serum estimates correlated with 24-h urine FCA in Cohort 1 (*ρ* = 0.56, *p* = 0.011, (**a**), but introduced significant bias in FCA estimates (**b**). In Cohort 2, we found no correlation between 1-h serum estimates of FCA and 24-h urine fractional calcium absorption (*ρ* = 0.40, *p* = 0.286, (**c**). Thus, testing of bias via Bland-Altman analysis (**d**) was not relevant

**Fig. 2:**
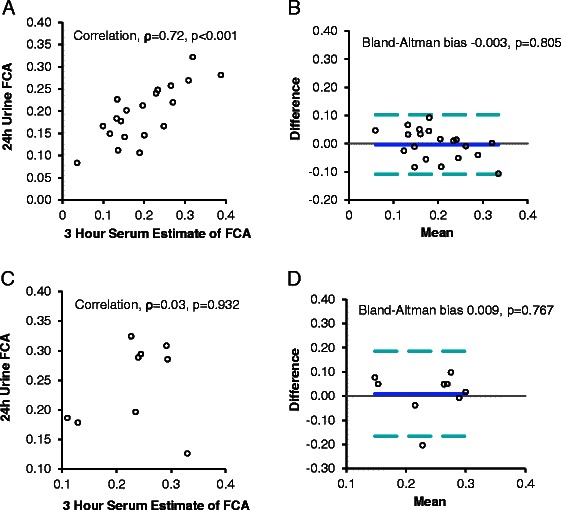
Three Hour Serum Isotope Estimates Compared to 24-h Urine Fractional Calcium Absorption (FCA) Values. Three-hour serum estimates correlated with 24-h urine FCA in Cohort 1 (*ρ* = 0.72, *p* < 0.001, (**a**) and values were not biased (**b**). However in Cohort 2, we found no correlation between 3-h serum estimates of FCA and 24-h urine FCA (*ρ* = 0.03, *p* = 0.932, (**c**). Thus, testing of bias via Bland-Altman analysis (**d**) was not relevant

**Fig. 3:**
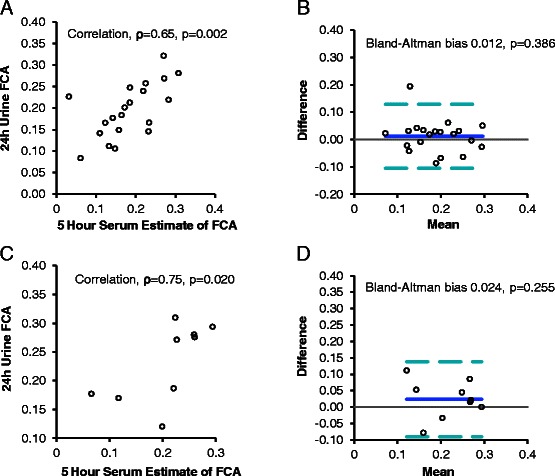
Five Hour Serum Isotope Estimates Compared to 24-h Urine Fractional Calcium Absorption (FCA) Values. Five-hour serum FCA estimates correlated with 24-h urine FCA in Cohort 1 (*ρ* = 0.65, *p* = 0.002, (**a**) and values were not biased (**b**). In Cohort 2, 5-h serum estimates of FCA correlated with 24-h urine fractional calcium absorption (*ρ* = 0.75, *p* = 0.020, **c**). Bias is illustrated in (**d**); the solid line represents the mean degree of bias and dashed lines indicate the 95 % limits of agreement. Bias was not statistically significant in Cohort 2, but 5-h serum estimates explained only 35 % of the variance in Cohort 2 24-h urine FCA values

The characteristics of Cohort 1 and Cohort 2 are summarized in Table 1. Based on outpatient food diaries, Cohort 1 consumed 856 ± 352 (mean ± SD) mg calcium per day and FCA (based on a 24-h urine collection) was 0.20 ± 0.06. FCA, based on the 24-h urine collection, correlated most closely with 3-h serum levels (ρ 0.72, *p* < 0.001) as compared to the 1-h (ρ 0.57, *p* = 0.008) and 5-h (ρ 0.65, *p* = 0.002) serum samples (Table 2; Figs. 1, 2, 3). There was no significant Bland-Altman bias between 24-h FCA and FCA estimated from 3- and 5-h serum levels. However, 1-h serum levels significantly overestimated 24-h urine FCA (bias +0.055, *p* < 0.001). In contrast to other reports [3, 8, 9], we found no improvement in correlation or variance, when serum levels were corrected for body surface area.

**Table 1:** Characteristics of subjects undergoing measurement of calcium absorption

	Cohort 1^a^	Cohort 2^b^	*p*-value
	*n* = 20	*n* = 9	
Age, years^c^	59 ± 7	60 ± 7	0.58
Race	18 White, 2 Black	5 White, 2 Black, 1 Hispanic, 1 Native American	
BMI, kg/m^2^	29 ± 5	36 ± 2	0.01
Calcium intake, mg/day	856 ± 352	1016 ± 453	0.31
Fractional Calcium Absorption	0.20 ± 0.06	0.23 ± 0.07	0.18
Serum Calcium, mmol/L	2.29 ± 0.09	2.26 ± 0.09	0.47
Serum Albumin, g/L	39 ± 2	36 ± 2	<0.001
Serum Magnesium, mmol/L	1.10 ± 0.13	1.08 ± 0.06	0.75
Serum Phosphorus, mmol/L	1.16 ± 0.15	1.00 ± 0.20	0.02
Serum 25(OH)D, nmol/L	54 ± 6	55 ± 11	0.70
Serum Parathyroid Hormone, ng/L	48 ± 19	43 ± 16	0.56
Serum Creatinine, μmol/L	77 ± 18	83 ± 13	0.44

^a^Cohort 1 was used to evaluate spot serum isotope approaches to measuring calcium absorption, using the dose-corrected levels of two stable calcium isotopes in a 24-h urine collection as the referent method

^b^Cohort 2 data was used to validate equations derived from Cohort 1

^c^All data exhibited a normal distribution and are expressed as mean ± standard deviation

**Table 2:** Relationship between gold-standard and new methods of measuring fractional intestinal calcium absorption in cohort 1

New method	FCA^a^	Correlation coefficient (*p*-value)	Bias^b^ (*p*-value)	Linear regression formula	R^2^	Bias^b^ (*p*-value)	RMSPE^c^
1 h Serum	0.14 ± 0.06	0.57 (0.008)	0.055 (<0.001)	Value x 0.5942 + 0.1119 = FCA	0.29	0 (0.999)	0.052
3 h Serum	0.20 ± 0.09	0.72 (<0.001)	-0.003 (0.805)	Value x 0.577 + 0.081 = FCA	0.58	0 (0.997)	0.040
5 h Serum	0.18 ± 0.07	0.65 (0.002)	0.012 (0.386)	Value x 0.541 + 0.096 = FCA	0.36	0 (0.997)	0.050

^a^“FCA” denotes fractional calcium absorption. Data are summarized using the mean ± SD. All new methods are compared to the referent method of measuring intestinal calcium absorption based on the dose-corrected ratio of dual stable isotopes in a 24-h urine collection, which equaled 0.20 ± 0.06 in Cohort 1 (*n* = 20)

^b^Bias was assessed using the Bland-Altman method, first reported for the raw data versus the referent values (column 4) and later reported for the derived formula versus the referent (column 7). A Bland-Altman test p-value >0.05 indicates that there is no significant difference between paired values using the gold-standard and the new method of measuring FCA

^c^“RMSPE” indicates the root mean square prediction error

We derived linear regression equations using serum tracer levels to predict 24-h urine FCA measurements. FCA determined from 3-h serum levels (FCA = Value x 0.577 + 0.081) explained the greatest variance in 24-h urine FCA levels (R^2^ 0.58, *p* < 0.001). By contrast, linear regression equations using 1-h and 5-h serum levels explained only 29 and 36 % of the variance in 24-h urine FCA, respectively. In multivariate analyses, incorporation of subjects’ GFR with their 3-h serum tracer measurement improved R^2^ to 0.61 (*p* < 0.001). Surprisingly, incorporation of subjects’ body surface area, body mass index, age, calcium intake, PTH and 25(OH)D levels did not improve ability to predict true FCA values.

We used Cohort 2 values to validate findings identified in Cohort 1. The characteristics of women in Cohort 2 were similar to those of Cohort 1 (Table 1) except for higher BMI (*p* = 0.01) and lower serum albumin (*p* < 0.001) and phosphorus (*p* = 0.02) levels. Cohorts 1 and 2 had similar FCA (Table 1). In Cohort 2, 24-h urine FCA correlated with 5-h serum estimates (ρ 0.75, *p* = 0.02) but not with 1-h estimates (ρ 0.40, *p* = 0.29) or 3-h estimates (ρ 0.03, *p* = 0.92) (Figs. 1, 2, 3). However, 5-h serum estimates explained only 35 % of the variance in 24-h urine FCA values.

Table 3 summarizes how well the best method identified in Cohort 1 (a linear regression equation using 3-h serum isotope values) predicted 24-h urine FCA values in Cohort 2. We found that 24-h FCA did not correlate with 3-h serum estimates entered into a linear regression equation, either with GFR (ρ 0.02, *p* = 0.97) or without GFR (ρ 0.03, *p* = 0.93). However, one outlier in Cohort 2 had a low 24-h urine FCA of 0.12. When we removed this subject’s data from analyses, we still found no significant correlation between the 24-h urine and 3-h serum FCA levels with GFR (ρ 0.45, *p* = 0.26) or without GFR (ρ 0.47, *p* = 0.23). Additionally, we detected a statistically significant bias, with overestimation of FCA when using the 3-h serum measurement (+0.043, *p* = 0.015) or the 3-h serum measurement and GFR (+0.047, *p* = 0.007).

**Table 3:** Assessment of Alternative Methods to Estimate Fractional Calcium Absorption in Cohort 2

New Method^a^	FCA^b^	Bias^c^ (*p*-value)	Linear Regression Equation	Correlation Coefficient, Calculated vs. Referent (*p*-value)	RMSPE^d^	Correlation Coefficient, Calculated vs. Referent^e^ (*p*-value)	Bias^c, e^ (*p*-value)	RMSPE^a, d, e^
3-h Serum	0.21 ± 0.04	0.009 (0.767)	Value x 0.577 + 0.081 = FCA	0.03 (0.932)	0.074	0.47 (0.233)	0.043 (0.015)	0.039
3-h Serum & GFR	0.21 ± 0.05	N/A	Value x 0.585 + 0.001 x GFR + 0.031 = FCA	0.02 (0.966)	0.071	0.45 (0.260)	0.047 (0.007)	0.037

^a^The two best methods identified in Cohort 1 were validated in Cohort 2

^b^“FCA” denotes intestinal fractional calcium absorption. Data are summarized using the mean ± SD. All new methods are compared to referent method of measuring intestinal calcium absorption based on the dose-corrected ratio of dual stable isotopes in a 24-h urine collection, which equaled 0.23 ± 0.07 in Cohort 2 (*n* = 9)

^c^Bias was assessed using the Bland-Altman method, first reported for the raw data versus the referent values (column 3) and then reported for the derived formula minus one outlier (column 8). A Bland-Altman test p-value >0.05 indicates that there is no significant difference between paired values using the gold-standard and the new method of measuring FCA

^d^“RMSPE” indicates the root mean square prediction error

^e^Data without one outlier who had low FCA (12 %)

## Discussion

Adequate calcium intake and absorption is a critical nutritional aspect of preventing or treating osteoporosis [19]. Postmenopausal women experience a 20-25 % decline in calcium absorption between the ages of 40 and 60 attributed to increasing age and estrogen deficiency [5]. As low calcium absorption is a risk factor for hip fracture [2], its measurement could be useful clinically. We evaluated whether FCA could be estimated by serum calcium tracer levels in postmenopausal women with vitamin D insufficiency. In Cohort 1, 3-h serum isotope levels were more accurate than 1-h or 5-h serum measurements, but only explained 58 % of the variance in true FCA. Unfortunately, we could not corroborate 3-h serum tracer levels as a valid method of measuring FCA in a second cohort of women with very similar characteristics.

Previous studies (Table 4) reported successful use of serum measurements to estimate FCA. Depending on the study, spot serum tracer levels explained 81 to 94 % of the variability in calcium absorption. Regrettably, the pre-defined “gold-standard” method for measuring FCA differed by study. Two studies [8, 9] compared spot serum isotope values to average FCA values obtained using three different “gold standard” methods: the oral to intravenous isotope ratio in a serum sample at 24 h, in a 24-h urine collection, and FCA based on kinetic modeling. A third study [3] defined the gold standard as the average value of tracer ratios (oral to intravenous) in multiple serum and urine samples collected over 6-10 days following tracer administration. A fourth study [10] used the ratio of cumulative recovery of oral to intravenous isotope in three consecutive eight hour urine collections as described by Yergey et al. [12]. In a fifth study [20], the gold standard method was the ratio of 5-h oral calcium specific activity to the 3-h intravenous calcium specific activity. None of the studies used Bland-Altman statistics to compare spot serum tracer estimates to gold-standard measures of FCA.

**Table 4:** Summary of studies comparing tracer methods to estimate fractional calcium absorption^a^

Subjects	Measures, New Method, Gold Standard Method (GSM)	Linear Regression Model to Estimate FCA^b^	Variance, *p*-value
265 women	554 measures, 5-h serum, GSM: average of oral to iv tracer valuesin multiple serum and urine samples collected for 6-10 days	^c^FCA = 2.3045(Serum) + 0.021953(Weight^0.425^) (Height^0.725^) - 0.1735	R^2^ 0.91
≥35 years old [3]			p not provided
30 men	New method at baseline and GSM 6 days later, 5-h serum, GSM: quotient of 5-h oral radioactivity to 3-h iv radioactivity	^c^FCA = (Serum)^0.92373^ x (0.385 x (Height^0.5285^) (Weight^0.3721^))	R^2^ 0.90
20-60 years old [20]			*p* < 0.001
12 men	24 measures, 5-h serum, GSM: average of oral to iv tracer values in multiple serum and urine samples collected at multiple times, and quotient of 5-h oral radioactivity to 3-h iv radioactivity	Not provided	R^2^ 0.92
36 ± 5 years old [21]			p not provided
19 men and women	19 measures, 4-h serum, GSM: ratio of cumulative recovery of oral to iv tracers in 24-h urine collection	^e^FCA = ratio of oral calcium tracer to intravenous tracer	R^2^ 0.83
≥50 years old [10]			*p* < 0.001
22 girls	31 measures, 4-h serum, GSM: Average of 3 values- oral to iv ratio in 24-h serum, 24-h urine and kinetic model	^c,d,f^FCA = 1.334(Serum)^0.7872^BSA^1.7132^e^(-0.01652PMA)^	R^2^ 0.94
10-15 years old [8]			*p* < 0.001
26 women	129 measures, 4, 5, 6 h serum, GSM: Average of 3 values- oral to iv ratio in 24-h serum, 24-h urine and kinetic model	^c^FCA = 1.3609(4 h Serum)^0.8703^BSA^0.8708^	R^2^ 0.78
19-67 years old [9]			*p* < 0.001
		^c^FCA = 1.4065(5 h Serum)^0.8437^BSA^0.7785^	R^2^ 0.78
			*p* < 0.001
		^c^FCA = 1.5828(6 h Serum)^0.8636^BSA^0.7142^	R^2^ 0.81
			*p* < 0.001

^a^Studies summarized in this table reported the amount of variance (R^2^) in fractional calcium absorption explained by a spot serum tracer level. Two other notable studies [12, 17] reported correlation coefficients only

^b^“FCA” denotes fractional calcium absorption

^c^In this formula, the serum level reflects the radioactivity or stable calcium isotope level as a fractional dose per mL, per gram/mL of serum calcium

^d^“BSA” refers to body surface area as defined by the DuBois equation [22]

^e^In this study, the oral tracer was given with breakfast and the intravenous tracer was given two hours later, with serum drawn 4 h after breakfast

^f^“PMA” refers to post-menarchal age

There are other reasons why our study disagrees with prior studies on whether a spot serum tracer level can accurately measure FCA. We examined FCA in post-menopausal women with vitamin D insufficiency, whereas previous studies recruited men [10, 20], adolescent or premenopausal women [8, 9] or postmenopausal women without regard to vitamin D status [3]. Several studies (Table 4) measured FCA multiple times in one individual; an approach that likely overestimates correlations between serum methods and gold-standard methods of estimating FCA. Whereas nearly all prior studies used correlation coefficients to compare different methods of measuring calcium absorption, the more rigorous Bland-Altman test to estimate bias was not utilized. Finally, only one study [8] validated their findings in a separate patient cohort.

Our study has several strengths. Our population of postmenopausal women arguably reflects a group of patients most likely to benefit from accurate measurement of FCA. We used the gold-standard approach to measure FCA and collected urine for 24 h during an inpatient hospital stay. We used the Bland-Altman test to assess potential bias between measurement methods, in addition to using correlation coefficients. We matched subjects’ inpatient meals to their usual outpatient nutritional intake. We assessed new measurement methods in a second cohort of women. Of course, our study has some weaknesses. We cannot state whether our results would apply to other groups of patients. While our study sample size was small, it is nearly identical to that of several other studies (Table 4). Given the poor correlation coefficients and significant bias using serum measurements, it seems very unlikely that our conclusions would be altered by studying more subjects.

## Conclusion

We conclude that serum tracer levels correlate with gold-standard FCA values, but cannot replace gold-standard FCA measurements. Additionally, even in the first cohort of women, the best spot serum isotope method only explained 58 % of the variance in FCA. We recommend continued use of dual calcium tracers and a subsequent 24-h urine collection for accurate measurement of FCA.

## Abbreviations

Ca: Calcium
CRU: Clinical research unit
FCA: Fractional calcium absorption
GFR: Glomerular filtration rate
HPLC: High performance liquid chromatography
MDRD: Modification of diet in renal disease
PTH: Parathyroid hormone
RMSPE: Root mean square prediction error
SD: Standard deviation
UW: University of Wisconsin
